# A CpG-Oligodeoxynucleotide Suppresses Th2/Th17 Inflammation by Inhibiting IL-33/ST2 Signaling in Mice from a Model of Adoptive Dendritic Cell Transfer of Smoke-Induced Asthma

**DOI:** 10.3390/ijms24043130

**Published:** 2023-02-04

**Authors:** Xuena Yang, Beiting Su, Jing Liu, Li Zheng, Peizhi Tao, Yusen Lin, Xiaoling Zou, Hailing Yang, Wenbin Wu, Ping Meng, Tiantuo Zhang, Hongtao Li

**Affiliations:** 1 Department of Pulmonary and Critical Care Medicine, The Third Affiliated Hospital of Sun Yat-sen University, Institute of Respiratory Diseases of Sun Yat-sen University, Guangzhou 510630, China; 2 Department of Pulmonary and Critical Care Medicine, The Seventh Affiliated Hospital of Sun Yat-sen University, Shenzhen 518000, China

**Keywords:** asthma, CpG-ODN, interleukin-33, ST2, dendritic cell

## Abstract

Tobacco smoke exposure is a major environmental risk factor that facilitates the development and progression of asthma. Our previous study showed that CpG oligodeoxynucleotide (CpG-ODN) inhibits thymic stromal lymphopoietin (TSLP)-dendritic cells (DCs) to reduce Th2/Th17-related inflammatory response in smoke-related asthma. However, the mechanism underlying CpG-ODN -downregulated TSLP remains unclear. A combined house dust mite (HDM)/cigarette smoke extract (CSE) model was used to assess the effects of CpG-ODN on airway inflammation, Th2/Th17 immune response, and amount of IL-33/ST2 and TSLP in mice with smoke-related asthma induced by adoptive transfer of bone-marrow-derived dendritic cells (BMDCs) and in the cultured human bronchial epithelium (HBE) cells administered anti-ST2, HDM, and/or CSE. In vivo, compared to the HDM alone model, the combined HDM/CSE model had aggravated inflammatory responses, while CpG-ODN attenuated airway inflammation, airway collagen deposition, and goblet cell hyperplasia and reduced the levels of IL-33/ST2, TSLP, and Th2/Th17-cytokines in the combined model. In vitro, IL-33/ST2 pathway activation promoted TSLP production in HBE cells, which could be inhibited by CpG-ODN. CpG-ODN administration alleviated Th2/Th17 inflammatory response, decreased the infiltration of inflammatory cells into the airway, and improved the remodeling of smoke-related asthma. The underlying mechanism may be that CpG-ODN inhibits the TSLP-DCs pathway by downregulating the IL-33/ST2 axis.

## 1. Introduction

Asthma is a common chronic respiratory disease with prevalence rates ranging from 1 to 18% in different countries [1]. It is a heterogeneous disease with various phenotypes. Among these, smoke-related asthma is a phenotype induced by allergens and exacerbated by tobacco smoke [2]. Tobacco smoke exposure is one of the major environmental risk factors for asthma that can aggravate the severity of clinical symptoms, increase the frequency of acute exacerbations, decrease lung function, and reduce the efficacy of current asthma treatments, consuming a large number of medical resources each year [3]. Both active and passive smoking are associated with the development and progression of asthma [4]. Active smoking is common in asthmatic individuals; the prevalence of active smoking in adult asthmatics from low- and middle-income countries is about 25%, which places them at an increased risk of severe symptoms and reduced response to steroid therapy [5]. Removal from the tobacco smoke environment improves the clinical symptoms and lung function of smoke-related asthma patients [6,7]. Despite aggressive tobacco control programs, the smoking cessation rate remains low. Therefore, developing other effective treatments for patients with smoke-related asthma is clinically significant.

Thymic stromal lymphopoietin (TSLP) is an essential modulator of asthma pathogenesis, mainly produced by human epithelial cells, keratinocytes, and bronchial smooth muscle cells [8,9]. Dendritic cells (DCs) are the main effector cells of TSLP [10]. In terms of the functional barriers of the respiratory system, DCs partake in the active biological response against foreign particulate antigens [11]. TSLP binds to TSLPR on DCs and stimulates the expression of the OX40 ligand. After DCs migrate to lymph nodes, the OX40 ligand interacts with OX40 on naive T cells to activate CD4^+^ T cell differentiation, promoting Th2 immune response [8]. In a previous study, we demonstrated that tobacco smoke induces TSLP secretion, DCs proliferation and maturation, and mediates naive T cell differentiation into Th2/Th17 subgroups in asthmatic mice [12]. These events could prompt the production of proinflammatory cytokines, such as Th2 type [interleukin (IL)-4, IL-5, and IL-13] and Th17 type (IL-17) inflammatory molecules, enhancing the production of immunoglobulin (Ig) E, mast cells and mucus, promotes the infiltration of eosinophils and neutrophils in the respiratory tract and increases airway hyperresponsiveness and hormone insensitivity [13].

IL-33 is one of the key cytokines involved in allergic airway diseases, along with TSLP and IL-25; together, these are referred to as epithelium-derived cytokines [14]. The IL-33 receptor ST2 belongs to the Toll-like receptor/interleukin-1 receptor (TIR) superfamily and has a high expression in DCs, macrophages, natural killer (NK) cells, and innate lymphoid group 2 cells (ILC2s) [15,16]. The soluble isoform of ST2 (sST2) might not be a signaling molecule but rather a decoy receptor binding IL-33, inhibiting its binding to membrane-bound ST2 and subsequent signaling [17]. House dust mites (HDM) are clinical allergens that trigger asthma worldwide, promoting IL-33 secretion by epithelial cells, and participating in the development and progression of allergic diseases [18]. The targeted inhibition or knockout of IL-33/ST2 can prevent asthma [19]. Reportedly, exposure to tobacco smoke increases the level of IL-33/ST2 in mice, causing airway inflammation and airway mucin production; these phenomena are suppressed by IL-33-neutralizing antibodies [20]. IL-33 also contributes to TSLP production through airway epithelial cells [21]. IL-33-neutralizing antibodies inhibit the production of TSLP in the airway in a murine model of ovalbumin (OVA)-induced allergic asthma [22]. Furthermore, IL-33 does not directly trigger the differentiation of CD4^+^ T cells into Th17 cells, suggesting that IL-33 might induce the differentiation of naive T lymphocytes by driving TSLP-DCs in upstream pathways [22]. Thus, it could be speculated that IL-33 drives DCs through TSLP to effectuate the reaction downstream of T lymphocytes.

Inhaled corticosteroids (ICS) are currently the major anti-inflammatory agents for the treatment of asthma. However, considering the high incidence of adverse effects associated with long-term high-dose ICS, the significant heterogeneity of smoke-related asthma increases the demand for ICS. The dose–response curve of medium/high-dose ICS showing a plateau effect prompts us to develop new control measures as an alternative treatment to ICS or use it in combination with ICS to reduce the dosage required for the prevention and treatment of smoke-related asthma [5]. A CpG oligodeoxynucleotide (CpG-ODN) is an artificially mimicked oligonucleotide fragment widely present in bacterial and viral DNAs [23]. It is an unmethylated deoxynucleotide fragment with a core CG sequence. As an immune adjuvant, CpG-ODN has potent immunomodulatory effects; this new formulation has shown efficacy in preliminary clinical studies of allergic asthma [24]. Our previous study showed that CpG-ODN administration alleviates the Th2/Th17 type inflammatory response to smoke-related asthma by inhibiting the TSLP-DC pathway [12]. Initially, we observed that CpG-ODN treatment affects the expression of IL-33 in smoke-related asthmatic mice. Therefore, we hypothesized that CpG-ODN administration suppresses the TSLP-DC pathway by downregulating the IL-33/ST2, thereby alleviating the Th2/Th17 type inflammatory response to smoke-related asthma.

## 2. Results

### 2.1. CpG-ODN Treatment Attenuates Airway Inflammation, Airway Collagen Accumulation, and Goblet Cell Hyperplasia in Mice with Smoke-Related Asthma Triggered by Adoptive Transfer of DCs

**Figure 1:**
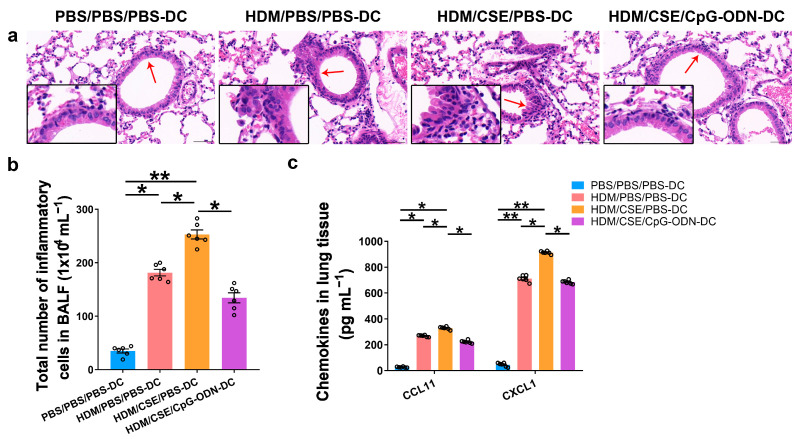
CpG-ODN inhibits airway inflammation in mice with smoke-related asthma. (**a**) Representative hematoxylin and eosin-stained lung tissue sections (×200). The red arrows point to the area shown at a higher magnification in the left lower quadrant. Scale bars: 50 μm. (**b**) Total number of inflammatory cells in BALF. (**c**) ELISA of CCL11 and CXCL1 in lung homogenates. All experiments were performed two times independently, with n = 6 mice/experiment and three technical replicates. Shapiro–Wilk tests were used to test for normality, and one-way ANOVA was used for multiple unmatched groups, followed by Sidak’s test for multiple comparisons. Data are mean ± s.e.m. * *p* < 0.05 and ** *p* < 0.01 vs. the counterpart group.

**Figure 2:**
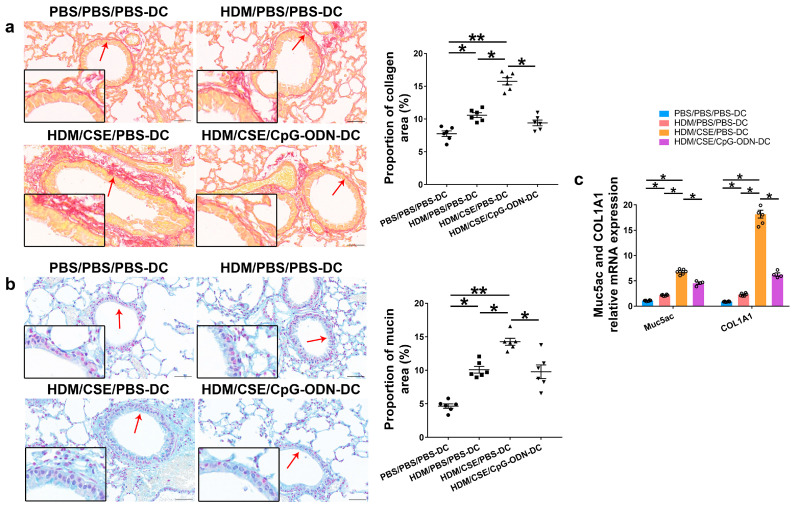
CpG-ODN inhibits airway remodeling in mice with smoke-related asthma. (**a**) Representative micrographs showing airway collagen deposition in Picro Sirius red-stained lung samples (×200). The red arrows point to the area shown at a higher magnification in the left lower quadrant. Scale bars: 50 μm. Proportion of collagen area derived as collagen-positive area/total bronchiole area in bronchioles of similar size using Image-Pro Plus 4.5 (Media Cybernetics, Inc., Rockville, MD, USA). (**b**) Representative micrographs showing mucin production in Alcian blue (pH 2.5)-stained lung sections (×200). The red arrows point to the area shown at a higher magnification in the left lower quadrant. Scale bars: 50 μm. Proportion of mucin area derived as mucin-positive area/total bronchiole area in bronchioles of similar size using Image-Pro Plus 4.5. (**c**) Relative Muc5ac and COL1A1 mRNA content was measured by qRT-PCR in lung tissue samples. All experiments were performed two times independently, with n = 6 mice/experiment and three technical replicates. Shapiro–Wilk tests were used to test for normality, and one-way ANOVA was used for multiple unmatched groups, followed by Sidak’s test for multiple comparisons. Data are mean ± s.e.m. * *p* < 0.05 and ** *p* < 0.01 vs. the counterpart group.

Based on our previous study, we used the adoptive transfer of BMDCs to establish a smoke-related asthma model and determine CpG-ODN function via the DCs pathway in regulating HDM/cigarette smoke extract (CSE)-induced lung inflammation in mouse models (Additional file 1: Figure S1). We injected phosphate-buffered saline (PBS)/PBS/PBS-pulsed, HDM/PBS/PBS-pulsed, HDM/CSE/PBS-pulsed, or HDM/CSE/CpG-ODN-pulsed BMDCs into C57BL/6 mice. After re-challenge with HDM or PBS, the HDM/CSE/PBS-DC group showed an elevated degree of inflammatory cell infiltration in peribronchial and perivascular tissues compared to the PBS/PBS/PBS-DC group (Figure 1a). Total inflammatory cell number in bronchoalveolar lavage fluid (BALF) (Figure 1b) and the expressions of eosinophil chemotactic factor (CCL11) and neutrophil chemotactic factor (CXCL1) in the lung homogenate (Figure 1c) were significantly higher in the HDM/PBS/PBS-DC group than in the PBS/PBS/PBS-DC group, but significantly lower than in the HDM/CSE/PBS-DC group. Consistently, in terms of airway reconstruction, collagen deposition (Figure 2a), mucin production (Figure 2b), and levels of airway remodeling indicators, including Mucin 5ac (Muc5ac) and Collagen type I alpha 1 (COL1A1) (Figure 2c) were significantly higher in the HDM/PBS/PBS-DC group than in the PBS/PBS/PBS-DC group, but significantly lower than in the HDM/CSE/PBS-DC group. Comparing the HDM/CSE/PBS-DC group with the HDM/CSE/CpG-ODN-DC group, all of the above processes could be inhibited by CpG-ODN in the smoke-related asthma model (Figure 1 and Figure 2). Taken together, these data demonstrated that smoke-related asthma exerts severe inflammatory responses and airway reconstruction, while CpG-ODN can suppress airway inflammation, airway collagen deposition, and mucin-producing cell hyperplasia through the DCs pathway in the mice model.

### 2.2. CpG-ODN Treatment Reduces the Levels of TSLP, Anti-HDM IgE, Proinflammatory Cytokines, and Th2/Th17-Cytokines in BMDCs Supernatants and Mice

**Figure 3:**
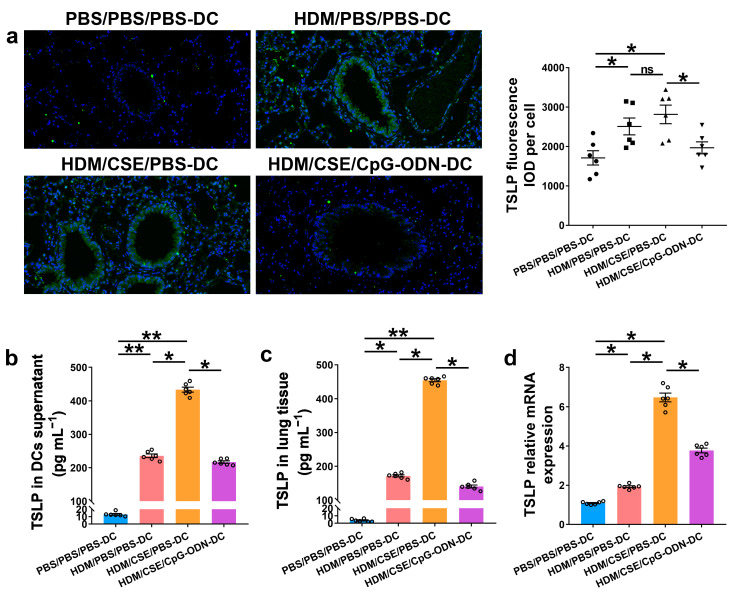
CpG-ODN treatment regulates TSLP in smoke-related asthma. (**a**) Representative images of TSLP (green) immunofluorescence of lung samples (×200). Scale bars: 50 μm. Semiquantitative protein expression of TSLP detected by immunofluorescence was determined as integral optical density (IOD) of TSLP (green)/number of cells. (**b**) TSLP protein level in BMDCs supernatants, assessed by ELISA. (**c**) TSLP protein content in lung homogenates, assessed by ELISA. (**d**) *TSLP* gene expression in lung homogenates, evaluated by qRT-PCR. All experiments were performed two times independently, with n = 6 mice/experiment and three technical replicates. Shapiro–Wilk tests were used to test for normality, and one-way ANOVA was used for multiple unmatched groups, followed by Sidak’s test for multiple comparisons. Data are mean ± s.e.m. * *p* < 0.05 and ** *p* < 0.01 vs. the counterpart group.

**Figure 4:**
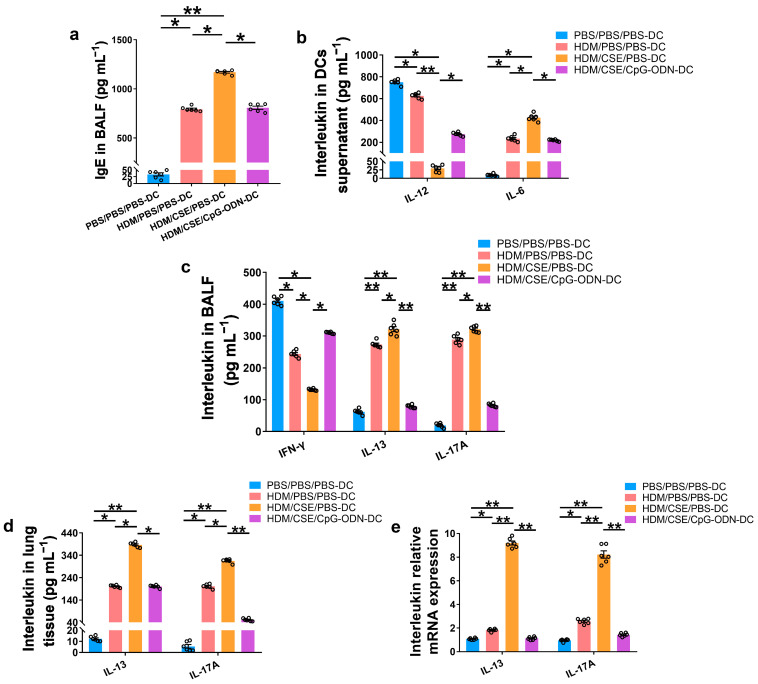
CpG-ODN treatment regulates anti-HDM IgE, pro-inflammatory, anti-inflammatory cytokines and Th1/Th2/Th17-cytokines in smoke-related asthma. (**a**) HDM-specific IgE amounts in BALF, assessed by ELISA. (**b**) IL-12 and IL-6 protein content in BMDCs supernatants, quantified by ELISA. (**c**) IFN-γ, IL-13, and IL-17A levels in BALF, as evaluated by ELISA. (**d**) IL-13 and IL-17A content in lung homogenates, evaluated by ELISA. (**e**) IL-13 and IL-17A gene expression in lung homogenates, evaluated by qRT-PCR. All experiments were performed two times independently, with n = 6 mice/experiment and three technical replicates. Shapiro–Wilk tests were used to test for normality, and one-way ANOVA was used for multiple unmatched groups, followed by Sidak’s test for multiple comparisons. Data are mean ± s.e.m. * *p* < 0.05 and ** *p* < 0.01 vs. the counterpart group.

To further verify whether CpG-ODN alleviates Th2/Th17-type inflammatory responses in smoke-related asthma by inhibiting the TSLP-DC pathway, consequently, the levels of TSLP, anti-HDM IgE, proinflammatory or anti-inflammatory cytokines, and Th1/Th2/Th17-cytokines were detected in the smoke-related asthma model induced by adoptive transfer of BMDCs. TSLP biosynthesis is associated with disease severity and occurs in structural and immune cells at the site of entry of allergens in the respiratory tract. Next, we assessed the levels of TSLP by immunofluorescence staining (Figure 3a), enzyme-linked immunosorbent assay (ELISA) (Figure 3b,c), and quantitative real-time polymerase chain reaction (qRT-PCR) (Figure 3d). The outcomes showed that the TSLP gene and protein expression levels were significantly increased in the HDM/CSE/PBS-DC group than in the PBS/PBS/PBS-DC and HDM/PBS/PBS-DC groups (Figure 3). Concurrently, HDM-specific IgE (Figure 4a) and IL-6 (Figure 4b) protein levels, and the mRNA and protein levels in type 2 inflammation represented by IL-13 (Figure 4c–e) and type 17 inflammation represented by IL-17A (Figure 4c–e) were significantly increased in the HDM/CSE/PBS-DC group compared to the PBS/PBS/PBS-DC and HDM/PBS/PBS-DC groups. On the other hand, IL-12 (Figure 4b) levels in BMDCs supernatants and type 1 inflammation represented by IFN-γ (Figure 4c) amounts in BALF were significantly decreased in the HDM/CSE/PBS-DC group compared to the PBS/PBS/PBS-DC and HDM/PBS/PBS-DC groups.

Compared HDM/CSE/PBS-DC group with HDM/CSE/CpG-ODN-DC group, CpG-ODN significantly reduced the levels of TSLP, anti-HDM IgE, IL-6, IL-13, and IL-17 (Figure 3 and Figure 4). On the other hand, it prevented the HDM/CSE-induced inhibition of IL-12 (Figure 4b) and induced IFN-γ (Figure 4c) expression in mice with smoke-related asthma triggered by the adoptive transfer of BMDCs. This finding indicated that the TSLP expression was exacerbated, which caused Th2/Th17 inflammation, while CpG-ODN suppressed TSLP levels and alleviated Th2/Th17 inflammation in the smoke-related asthma murine model.

### 2.3. CpG-ODN Treatment Reduces IL-33/ST2 in BMDCs Supernatants and Mice

**Figure 5:**
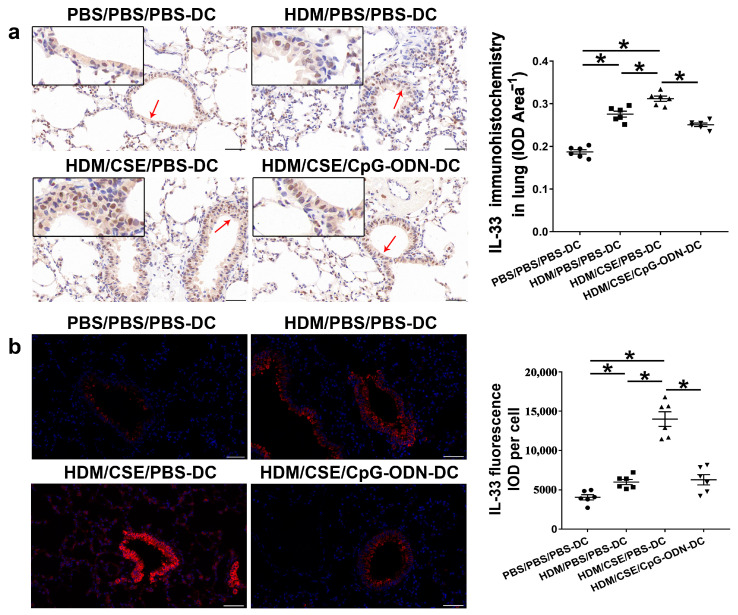
IL-33 in lung samples is reduced by CpG-ODN treatment. (**a**) Representative micrographs after immunohistochemical staining of IL-33(×200). The red arrows point to the area shown at a higher magnification in the left lower quadrant. Scale bars: 50 μm. IL-33 signals appeared as brown spots. IOD of IL-33 was calculated by Image-Pro Plus 4.5. The positivity rate of IL-33 was determined as IOD/total bronchiole area. (**b**) Representative micrographs of IL-33 (red) immunofluorescence in lung sections (×200). Scale bars: 50 μm. IOD of IL-33 was calculated by Image-Pro Plus 4.5. Semiquantitative protein content of IL-33 detected by immunofluorescence, as assessed as IOD of IL-33 (red)/number of cells. All experiments were performed two times independently, with n = 6 mice/experiment and three technical replicates. Shapiro–Wilk tests were used to test for normality, and one-way ANOVA was used for multiple unmatched groups, followed by Sidak’s test for multiple comparisons. Data are mean ± s.e.m. * *p* < 0.05 vs. the counterpart group.

**Figure 6:**
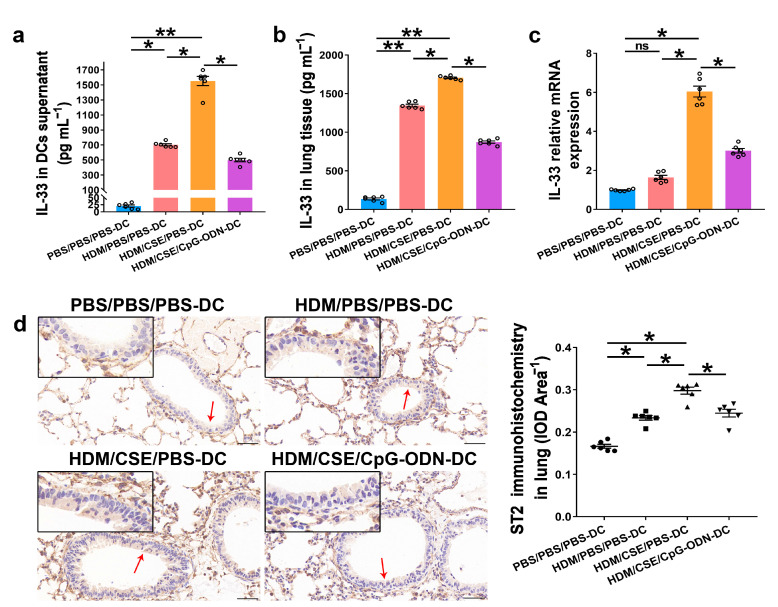
IL-33/ST2 levels in BMDCs supernatants and lung samples are reduced by CpG-ODN treatment. (**a**) IL-33 level in BMDCs supernatants, examined by ELISA. (**b**) IL-33 level in lung homogenates, evaluated by ELISA. (**c**) *IL-33* gene expression in lung homogenates, examined by qRT-PCR. (**d**) Representative micrographs after immunohistochemical staining of ST2 (×200). The red arrows point to the area shown at a higher magnification in the left lower quadrant. Scale bars: 50 μm. ST2 signals appeared as brown spots. IOD of ST2 was calculated by Image-Pro Plus 4.5. The positivity rate of ST2 was determined as IOD/total bronchiole area. All experiments were performed two times independently, with n = 6 mice/experiment and three technical replicates. Shapiro–Wilk tests were used to test for normality, and one-way ANOVA was used for multiple unmatched groups, followed by Sidak’s test for multiple comparisons. Data are mean ± s.e.m. * *p* < 0.05 and ** *p* < 0.01 vs. the counterpart group.

IL-33/ST2 is a key player in allergic airway diseases. Tobacco smoke elevates the level of IL-33/ST2 in mice, causing airway inflammation and mucin expression in the airway. To examine the correlation between CpG-ODN treatment and IL-33/ST2 expression, we quantitated the IL-33/ST2 levels. The levels of IL-33 mRNA and protein reflected by immunohistochemistry (Figure 5a), immunofluorescence staining (Figure 5b), ELISA (Figure 6a–b), and qRT-PCR (Figure 6c) were significantly higher in the HDM/CSE/PBS-DC group than in PBS/PBS/PBS-DC and HDM/PBS/PBS-DC groups. Similar to IL-33, the ST2 levels had the same change between these groups, as examined by immunohistochemistry (Figure 6d). Moreover, after CpG-ODN treatment, mice in the HDM/CSE/CpG-ODN-DC group had significantly decreased IL-33 and ST2 mRNA and protein levels compared to the animals in the HDM/CSE/PBS-DC group (Figure 5 and Figure 6). These data suggested that the smoke-related asthma murine model has more expression of IL-33 and ST2, which is downregulated by CpG-ODN.

### 2.4. IL-33/ST2 Combination Promotes the Production of TSLP in HBE Cells and Is Inhibited by CpG-ODN Treatment

**Figure 7:**
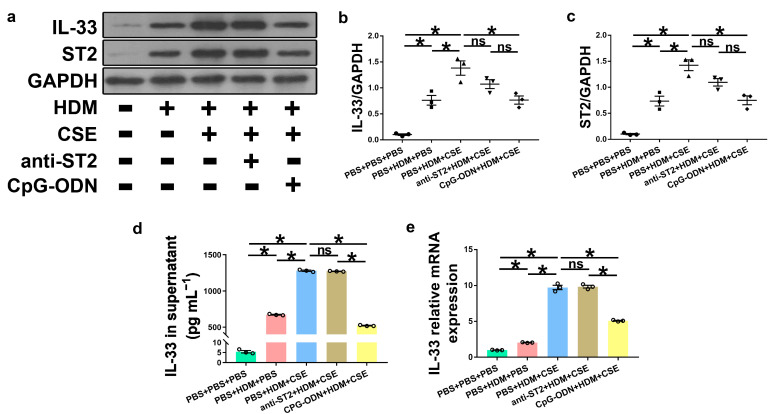
CpG-ODN treatment inhibits IL-33/ST2 upregulation in HBE cells. (**a**) Western blotting detection of IL-33/ST2 against GAPDH (utilized for normalization). (**b**) IL-33/GAPDH ratio was determined. (**c**) ST2/GAPDH ratio was determined. (**d**) IL-33 levels in HBE cell culture supernatants, examined by ELISA. (**e**) Relative IL-33 mRNA expression in HBE cells evaluated by qRT-PCR. All experiments were performed two times independently, with n = 3 per experiment and three technical replicates. Shapiro–Wilk tests were used to test for normality, and one-way ANOVA was used for multiple unmatched groups, followed by Sidak’s test for multiple comparisons. Data are mean ± s.e.m. * *p* < 0.05 vs. the counterpart group.

**Figure 8:**
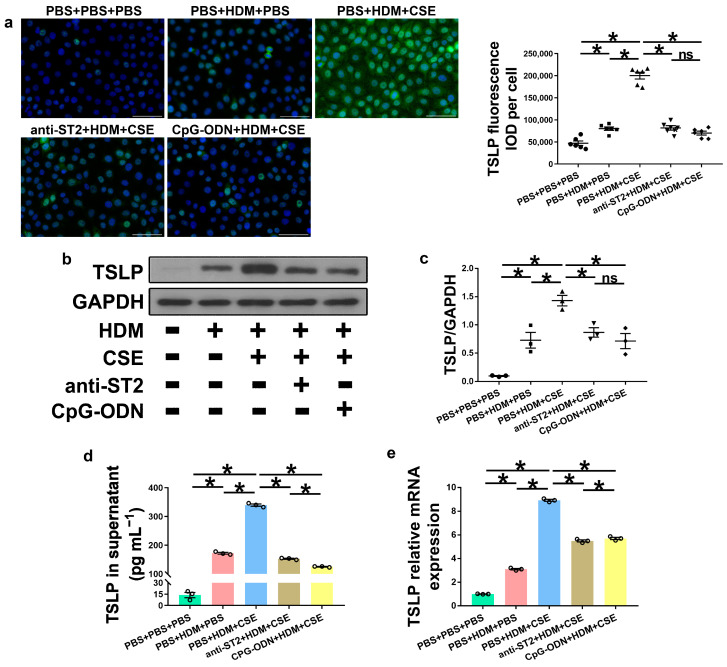
CpG-ODN treatment decreases TSLP expression in HBE cells. (**a**) Representative images of TSLP (green) immunofluorescence of HBE cells (×200). Scale bars: 50 μm. Semiquantitative protein expression of TSLP detected by immunofluorescence, determined as IOD of TSLP (green)/number of cells. (**b**) Western blotting detection of TSLP against GAPDH (utilized for normalization). (**c**) TSLP/GAPDH ratio was determined. (**d**) TSLP amounts in HBE cell culture supernatants, examined by ELISA. (**e**) Relative TSLP mRNA expression in HBE cells, evaluated by qRT-PCR. All experiments were performed two times independently, with n = 3 per experiment and three technical replicates. Shapiro–Wilk tests were used to test for normality, and one-way ANOVA was used for multiple unmatched groups, followed by Sidak’s test for multiple comparisons. Data are mean ± s.e.m. * *p* < 0.05 vs. the counterpart group.

IL-33, TSLP, and IL-25 are collectively referred to as epithelium-derived cytokines. To further examine the correlations among CpG-ODN, IL-33/ST2, and TSLP, we performed an in vitro assessment of HBE cells. Compared to PBS administration alone, HBE cells with HDM administration showed more IL-33/ST2 mRNA and protein, as examined by Western blotting (Figure 7a–c), ELISA (Figure 7d), and qRT-PCR (Figure 7e). Similar to IL-33/ST2, TSLP levels were examined by using immunofluorescence staining (Figure 8a), Western blotting (Figure 8b,c), ELISA (Figure 8d), and qRT-PCR (Figure 8e), which showed a similar change trend between the PBS + PBS + PBS and PBS + HDM + PBS groups. After co-exposure to HDM and CSE, HBE cells showed more expression of IL-33/ST2 and TSLP than HBE cells with the administration of HDM alone (Figure 7 and Figure 8). Compared anti-ST2 + HDM + CSE group with PBS + HDM + CSE group, we found that pretreatment of HBE cells with anti-ST2 neutralizing Abs significantly declined the mRNA and protein levels of TSLP (Figure 8) but did not have a significant effect on levels of IL-33 and ST2 (Figure 7). The administration of neutralizing Abs did not inhibit the expression but acted against the signaling pathway of IL-33/ST2 and then downregulated the expression of TSLP. Compared CpG-ODN + HDM + CSE group with PBS + HDM + CSE group, the pretreatment of HBE cells with CpG-ODN not only inhibited the expression of IL-33/ST2 but also effectuated the signaling pathway, thereby decreasing the expression of TSLP (Figure 7 and Figure 8). Compared to the anti-ST2 + HDM + CSE group, although the levels of IL-33/ST2 examined by Western blotting (Figure 7a–c) and levels of TSLP examined by immunofluorescence staining (Figure 8a) did not differ significantly, the levels of IL-33 examined by ELISA and qRT-PCR (Figure 7d,e) and TSLP examined by ELISA and qRT-PCR (Figure 8d,e) were significantly decreased in the CpG-ODN + HDM + CSE group. At the cellular level of HBE, CpG-ODN strongly downregulated TSLP more than anti-ST2. These results indicated that IL-33/ST2 upregulates TSLP, while CpG-ODN inhibits this process.

## 3. Discussion

In a previous study, we demonstrated that DCs exert a critical effect in smoke-related asthma [12]. In patients with COPD, CD40 expression in lung-resident DCs is high [25]. CSE stimulates BMDCs directly by augmenting CD40 expression and promoting naive CD4^+^ T cell differentiation into Th17 cells [26,27]. To clarify the direct role of DCs in smoke-related asthma, the principal advantage of this study was that the adoptive transfer of BMDCs was used to establish the murine smoke-related asthma model. This model presented a mixed inflammatory response of eosinophils and neutrophils, with Th2/Th17 polarization, which was consistent with our previous finding [12], indicating that DCs play a major role in smoke-related asthma; thus, it could be deduced that our model was established successfully. A potential limitation may be that airway hyperresponsiveness (AHR) was not assessed in this study because whole-body plethysmography was unavailable because of the COVID-19 pandemic. In our previous study, we successfully identified AHR in a smoke-related asthma model [12,28].

TSLP represents an essential immune modulator after exposure to environmental stressors, such as allergens, viruses, and pollutants, initiating a range of downstream inflammatory responses that consist mainly of Th2-type responses [29]. Moreover, TSLP is crucial in asthma-related inflammation in humans and mice, showing elevated levels in mouse models with antigen-related airway inflammatory disease; lung TSLP correlates with eosinophilia severity [30]. Consistent with the previous findings [31,32], our results showed that activated DCs express ST2 receptors on the surface and secrete IL-33 and TSLP. Additionally, TSLP promotes Th2 type inflammatory response and induces Th17 type inflammatory response. Therefore, it could be deduced that TSLP and IL-17A are involved in asthma development. In addition, previous findings suggested that TSLP induces Th17 cell differentiation in some pathologies [33,34]. In 2009, Tanaka et al. found that via DCs activation, human TSLP promotes differentiation in Th17 cells with the central memory T cell phenotype under Th2-polarizing conditions [35]. In the occupational asthma mouse model induced by toluene-diisocyanate, Th17 cell differentiation and IL-17 secretion are inhibited by intraperitoneally administered TSLP-Ab [36]. Consequently, asthma and airway inflammation were relieved. Nevertheless, how the Th17 inflammatory response is promoted by TSLP remains undefined.

IL-33 belongs to the IL-1 family and constitutes a proinflammatory cytokine secreted by damaged cells under attack by stressors, including allergens, viruses, and smoke [37,38,39]. In humans, IL-33 shows a constitutive expression in various tissues in specific cell types, including endothelial, epithelial, smooth muscle, and fibroblast cells [40]. In mice, IL-33 is highly expressed in the epithelial barrier, lymphoid organs, the brain, embryos, and inflamed tissues, such as the lung or the liver [41]. Activated DCs and macrophages represent the hematopoietic cell types with low IL-33 mRNA expression, while it is highly expressed in murine DCs and macrophages [42]. The IL-33/ST2 interaction activates Th2-mediated immunity and induces the accumulation of several proinflammatory cytokines. IL-33 and ST2 are also upregulated in COPD [43]. Interestingly, cigarette smoke, a major risk factor for COPD, upregulates IL-33 in epithelial, endothelial, and peripheral blood mononuclear cells. In individuals with concurrent allergic asthma and rhinitis, plasma IL-33 and IL-17 levels are elevated and positively correlated, suggesting the IL-33/ST2 axis might induce Th2 and Th17 immune responses in allergic airway diseases [44]. Park et al. reported that IL-33 has no direct function in naive CD4^+^ T cell differentiation to Th17 cells, but IL-33-treated DCs increase the surface expression of proteins that induce T cell activation and promote Th17 cell response through DC-produced IL-6 [45]. However, the mechanism of IL-33-promoted DCs maturation is yet unclear. HBE cells are the top source of IL-33 and TSLP expression in the human body, and IL-33 and TSLP levels are much higher in epithelial cells compared to DCs. To better conform to the occurrence of smoked-related asthma in the human population and further verify the correlation between IL-33 and TSLP, cell experiments were performed. As shown by Huang et al., TSLP expression is decreased after the use of IL-33 antagonists in the rat nasal mucosal epithelium; our in vitro assessment of HBE cells corroborated the previous findings that the expression of TSLP was reduced in response to anti-ST2 antibody [46]. These results suggested that IL-33 induces Th2/Th17 cell differentiation through the TSLP-DC pathway in both cell culture and animal models. In our previous study [12,28], we included CSE alone control in both the in vivo and in vitro model systems, which revealed that CSE aggravated lung inflammatory cell infiltration, airway remodeling, and mucus production in airway epithelium compared to the PBS control. Furthermore, to evaluate the effects of BMDCs on smoke-related asthma and to elucidate the underlying mechanisms by which CpG-ODN restores lung inflammation DC-related Th2/Th17 inflammatory response via IL-33/ST2 pathway, we did not perform CSE alone control in the current study. In the subsequent study, we would include the CSE alone control to determine if IL-33 is inhibited by CpG-ODN in the HDM or CSE arm or both.

DCs represent major players in both stages of asthma, partially because they can recognize the pathogen recognition receptors, including Toll-like receptors (TLRs), and identify the associated molecular patterns in allergens [47,48]. Among these, TLR9 has a critical immunomodulatory function in certain types of asthma. It induces substantial proinflammatory and Th1-biased responses instead of Th2-type or Th17-type immune responses [49,50,51]. CpG-ODN is a TLR9 agonist and a vaccine adjuvant for activating innate immunity and directly and indirectly regulating adaptive immunity [23]. In human, CpG-ODN can stimulate TLR9 to induce high interferon (IFN)-α production from plasmacytoid dendritic cells and prevents ILC2-driven Th2 responses [51], while murine bone marrow myeloid DCs produces IL-12 after activation of TLR9 by CpG-ODN and suppress Th2-type responses [52]. A previous study demonstrated that CpG-ODN treatment alleviates Th2/Th17 inflammatory response in asthmatic animals exposed to tobacco by inhibiting the TSLP-DC pathway [12]. However, the mechanism by which CpG inhibits the IL-33/ST2 pathway and further downregulates TSLP remains poorly understood. It was shown that CpG ODN-induced innate and adaptive IFN-γ production is dependent on IL-12 and MyD88 signaling by conventional dendritic cells (cDCs) and alveolar macrophages (AMs) [53]. CpG A can lead to the production of IFN-α driven IFN-γ to inhibit ILC2 function through a STAT1-dependent mechanism and ultimately inhibits IL-33-mediated AHR and airway inflammation [51]. Furthermore, in immune allergic responses, B-type CpG-DNA markedly suppressed poly(I:C)-induced TSLP production via the phosphorylation of c-Jun N-terminal kinase (JNK) [54]. As one of the reported TLR signaling suppressors, ST2L (a transmembrane receptor for IL-33) shows high levels on diverse hematopoiesis-derived cells under allergy conditions [42,45]. Upon activation, ST2L interacts with MyD88, suppressing CpG-induced TLR signaling pathways. Here, CpG-ODN administration markedly downregulated IL-33/ST2 and decreased inflammation in the smoke-related asthma model induced by the adoptive transfer of DCs. These findings indicated that CpG-ODN treatment might downregulate IL-33/ST2, thereby reducing the expression of TSLP, inhibiting the TSLP-DC pathway, alleviating Th2/Th17 inflammatory response in smoke-related asthma, and reducing the degree of airway inflammatory cell infiltration and remodeling. Our previous study showed that the combination of CpG-ODN and corticosteroid synergistically ameliorated corticosteroid insensitivity, airway eosinophilia, and neutrophilic inflammation in both allergic diseases and smoke-related asthma [12,28,55]. Additionally, some studies demonstrated that IL-33 and TSLP could regulate basophil development and peripheral basophilia [16,56,57]. In the current study, CpG-ODN treatment reduced the levels of TSLP and IL-33 in smoke-related asthma. It gives us an inspiration that the adoptive transfer of CpG-ODN treatment with HDM/CSE pulsed BMDCs may suppress the activities of basophils in smoke-related asthma; we would like to further clarify this question in our future study. A clinical trial revealed that CpG-ODN associated with virus-like particles (VLP) consisting of the bacteriophage Q beta coat protein as an efficient adjuvant shows clinical efficacy, safety, and tolerability in mild-to-moderate persistent allergic asthma [24]. Hence, it could be hypothesized that CpG-ODN has potential therapeutic benefits for asthma. Smoke-related asthma is characterized by intolerance to corticosteroids, making clinical treatment challenging. If CpG-ODN can attenuate the adverse outcomes of smoke-related asthma, patients will benefit from the approach, and it would constitute a potent novel therapeutic candidate for smoke-related asthma. To gain an in-depth insight into the function of CpG-ODN in smoke-induced asthma, a downstream study focusing more on the treatment effect rather than the prevention effect is essential. This would facilitate the investigation of the clinical efficacy of CpG-ODN in smoke-related asthma.

## 4. Materials and Methods

### 4.1. Mice

Specific pathogen-free (SPF) female C57BL/6 mice (6–8 weeks old), provided by Beijing Vital River Animal Technology (Beijing, China), were fed a standard sterile diet with free access to water under constant temperature (21 ± 2 °C) and humidity (30 ± 10%) under a 12 h/12 h light-dark cycle.

### 4.2. BMDCs Isolation and Treatments

In this study, BMDCs were obtained as described previously [12]. BMDCs were cultured in 24-well plates at a density of 1 × 10^6^ cells mL^−1^ in RPMI-1640 (HyClone, Logan, UT, USA) containing heat-inactivated fetal bovine serum (FBS, 10%, HyClone), penicillin and streptomycin (50 U mL^−1^ each, HyClone), GM-CSF (20 ng mL^−1^, PeproTech, Rocky Hill, CT, USA), and IL-4 (10 ng mL^−1^, PeproTech). About 3/4th of the culture medium was replaced with fresh medium and replenished with cytokines every 2 days. At 8 days, bone marrow-derived dendritic cells were enriched by positive selection with anti-CD11c magnetic beads (Miltenyi Biotec, Bergisch Gladbach, Germany) according to the manufacturer’s instructions. The enriched DCs were typically of >83% purity, as determined by flow cytometry. Then, the BMDCs were assigned to four groups. (1) In the control group (PBS/PBS/PBS-DC), the BMDCs were administered PBS (HyClone) with subsequent PBS administration; (2) In the HDM group (HDM/PBS/PBS-DC), the BMDCs were administered HDM (30 µg mL^−1^, Greer Laboratories, Lenoir, NC, USA) with subsequent PBS treatment; (3) In the HDM/CSE group (HDM/CSE/PBS-DC), the BMDCs were administered HDM (30 µg mL^−1^) and CSE (10 μL mL^−1^) with subsequent PBS treatment. CSE was prepared as described previously [28]. (4) In the HDM/CSE/CpG-ODN group (HDM/CSE/CpG-ODN-DC), the BMDCs were administered HDM (30 µg mL^−1^) and CSE (10 μL mL^−1^), followed by CpG-ODN (1 μg mL^−1^, Sangon Biotech, Shanghai, China) treatment. After consulting the relevant literature and conducting corresponding preliminary experiments, we selected the above concentrations of HDM, CSE, and CpG-ODN, which only affected the viability without inactivating the BMDCs [58,59,60]. The cells were incubated in a humid environment with 5% CO_2_ at 37 °C for 24 h after the above treatments. On day 9, supernatants in different groups were collected to detect the levels of IL-33, TSLP, IL-12p70, and IL-6. The ELISA results of the levels of IL-12p70 and IL-6 in the BMDC supernatants reflected the activation status of the cells. The BMDC viability ratio was 95%, as assessed by trypan blue staining after 24 h post-treatment.

### 4.3. Mouse Models of Smoke-Related Asthma Induced by Adoptive Transfer of BMDCs

The smoke-related asthma murine model triggered by the adoptive transfer of BMDCs was established, as reported by Han et al., with minor modifications [61]. On day 0, the animals were given anesthesia. Then, intratracheal instillation was performed with a fine metal catheter connected to a microinjector. Next, 10^6^ BM-derived cells from the four groups were instilled into the tracheas of anesthetized recipients. On days 10–12, the mice were challenged on 3 consecutive days intranasally with 10 μg of HDM (in 40 μL PBS). On day 13, euthanasia was carried out for BALF acquisition, lung homogenate preparation and lung tissue sectioning. A schematic diagram of the smoke-related asthma mouse model induced by the adoptive transfer of DCs is illustrated in Additional file 1: Figure S1. The protocol was approved by the Committee on the Ethics of Animal Experiments of the Third Affiliated Hospital of Sun Yat-sen University.

### 4.4. HBE Cell Culture and Treatment

HBE cells were purchased from Guangzhou Cellcook Biotechnology (Guangzhou, Guangdong, China), and authentication was performed by short tandem repeat (STR) DNA profiling (Biowing Applied Biotechnology, Shanghai, China). HBE cells were cultured in MEM with 10% FBS in a CO_2_ incubator (5% CO_2_ with saturated humidity at 37 °C) and assigned to five groups. (1) In the control group (PBS + PBS + PBS), the cells were pretreated with PBS for 1 h and then administered PBS for 8 h; (2) in the HDM group (PBS + HDM + PBS), HBE cells were pretreated with PBS for 1 h, followed by HDM (400 U mL^−1^) for 8 h; (3) in the HDM/CSE group (PBS + HDM + CSE), HBE cells were pretreated with PBS for 1 h, and then with HDM (400 U mL^−1^) and CSE (2.5%) for 8 h; (4) in the Anti-ST2 Ab/HDM/CSE group (anti-ST2 + HDM + CSE), HBE cells underwent pretreatment with anti-ST2 Ab (i.e., sST2, 10 μg mL^−1^, R&D Systems, Minneapolis, MN, USA) for 1 h, and then HDM (400 U mL^−1^) and CSE (2.5%) were administered for 8 h; (5) in the CpG-ODN/HDM/CSE group (CpG-ODN + HDM + CSE), HBE cells underwent CpG-ODN (5 × 10^−6^ M) pretreatment for 1 h, followed by the addition of HDM (400 U mL^−1^) and CSE (2.5%) for 8 h. The treatment doses for HDM and anti-ST2 Ab were acquired from previous successful experiments, with no cytotoxicity detected in the current preliminary experiments [62,63]. CpG-ODN and CSE doses were obtained from our preliminary experiments [25]. Subsequently, the supernatants and cells were collected to detect the levels of IL-33, TSLP, and ST2.

### 4.5. Preparation of BALF and Characterization of Cellular Infiltrates

BALF preparation and inflammatory cells were quantified in the BALF samples from the treated mice, as described previously [64].

### 4.6. Preparation of Lung Homogenates

The lung tissue underwent homogenization with 1% Triton X100 (Sigma Aldrich, St. Louis, MO, USA) in PBS supplemented with Complete Mini protease inhibitors (Roche, New York, NY, USA) utilizing a Precellys 24 tissue homogenizer (Bertin Technologies, France). The supernatants were collected by centrifugation (14,000 rpm for 10 min) and stored at −20 °C. A Pierce BCA protein assay kit was utilized for protein quantitation, used as per the manufacturer’s instructions (Thermo Fisher Scientific, Waltham, MA, USA). The samples were adjusted to 1 mg mL^−1^ protein before cytokine and chemokine level assessments.

### 4.7. ELISA

IL-33, TSLP, IL-12p70, and IL-6 levels in BMDCs supernatants; HDM-specific IgE, IFN-γ, IL-13, and IL-17A in BALF samples; IL-33, TSLP, IL-13, IL-17A, CCL11 and CXCL1 in lung homogenates; IL-33 and TSLP in HBE cell supernatants were measured using ELISA kits (HDM-specific IgE, Sigma: St. Louis, Missouri, USA; IL-33, TSLP, IFN-γ, IL-13, IL-17A, CCL11, and CXCL1, Bioss Inc., Woburn, MA, USA), as directed by the manufacturers

### 4.8. qRT-PCR

The total RNA was obtained from lung specimens and HBE cells using TRIzol reagent (Invitrogen, Carlsbad, CA, USA). Then, the mRNA levels of IL-33, TSLP, IL-13, IL-17A, Muc5ac, and Col1a1 in lung specimens and IL-33 and TSLP in HBE cells were quantitated by using qRT-PCR on an ABI PRISM^®^ 7500 System (Foster City, CA, USA). qRT-PCR primers specific for mouse IL-33, TSLP, IL-13, IL-17A, Muc5ac, Col1a1, and GAPDH mRNAs are listed in Additional file 1: Table S1.qRT-PCR primers specific for HBE IL-33, TSLP and GAPDH mRNAs are listed in Additional file 1: Table S2. GAPDH was utilized for normalization, and the 2^−ΔΔCt^ method was applied for data analysis.

### 4.9. Western Blotting

Immunoblotting was carried out using primary antibodies targeting IL-33, TSLP, and ST2 (Invitrogen) in HBE cells. The antibodies were diluted at a ratio of 1:1000.

### 4.10. Histology, Immunohistochemistry and Immunofluorescence

Lung fixation was conducted using 10% formalin at 20 cm H_2_O. After paraffin embedding, the tissues were sectioned to a 3 μm thickness and stained with hematoxylin and eosin, Alcian blue (pH 2.5), and Picro Sirius red, respectively. Immunohistochemistry was carried out using anti-IL-33 (Invitrogen) and anti-ST2L (Invitrogen) antibodies at a dilution of 2 μg mL^−1^ for lung samples. Immunofluorescence staining was carried out using anti-IL-33 (Invitrogen) and anti-TSLP (Invitrogen) in the lung samples, and anti-TSLP (Invitrogen) was used for the HBE cells; the antibodies were used at a concentration of 20 μg mL^−1^.

### 4.11. Statistical Analysis

Data are mean ± s.e.m. Shapiro–Wilk tests were used to test for normality, and one-way analysis of variance (ANOVA) was performed for multiple unmatched groups, followed by Sidak’s test for multiple comparisons. GraphPad Prism 7.0 (GraphPad Software, San Diego, CA, USA) was utilized to analyze all of the data. *p* < 0.05 indicated a statistically significant difference.

## Data Availability

The datasets used and/or analyzed during the current study are available from the corresponding author upon reasonable request.

## Supplementary Materials

The following supporting information can be downloaded at: https://www.mdpi.com/article/10.3390/ijms24043130/s1, Table S1: Sequence of primers for real-time PCR for mouse; Table S2: Sequence of primers for real-time PCR for HBE; Figure S1: Experimental protocol for the study.
