# Supplementary material for: A CpG-Oligodeoxynucleotide Suppresses Th2/Th17 Inflammation by Inhibiting IL-33/ST2 Signaling in Mice from a Model of Adoptive Dendritic Cell Transfer of Smoke-Induced Asthma
Source: Int J Mol Sci. 2023 Feb 4;24(4):3130. doi: 10.3390/ijms24043130 (PMC9962992; doi:10.3390/ijms24043130)
Supplement: Supplementary file 1 [file ijms-24-03130-s001.zip › ijms-2131253-supplementary.pdf]

Table S1. Sequence of primers for real-time PCR for mouse

Gene	**5'to 3' Sequence**	Size
IL-33	CAAGCATTTGCTGCGTCTGT	116 bp
	TTCTCCAAAACAAAACTAAC	
TSLP	CCTTCCTCAGCACACAATTG	170 bp
	TGATAAGCTGGCTCACAGAA	
IL-13	ACCCAACAACCACCTATGCT	150 bp
	TGCACTCATTGGTGGAGGTA	
Muc5ac	TGACCAAGAGCTCCGTCTTA	122 bp
	GAAGAGCAGCCCCAGCCTAG	
COL1A1	TGGCCCTCCTGGCAAGAATG	104 bp
	TTCCAGGCAATCCACGAGC	
IL-17A	TCATCCCTCAAAGCTCAGCG	112 bp
	CTTCTGGAGCTCACTTTTGC	
GAPDH	CCTGGATACCGCAGCTAGGA	112 bp
	GCGGCGCAATACGAATGCCCC	

Table S2. Sequence of primers for real-time PCR for HBE

Gene	**5'to 3' Sequence**	Size
IL-33	AACAGCAGTCTACTGTGGAG	151 bp
	TCAACACCGTCACCTGATT	
TSLP	GCAATCGGCCACATTGCCTT	136 bp
	TCCGAATAGCCTGGGCACCA	
GAPDH	GCTCATTTGCAGGGGGGAG	138 bp
	GTTGGTGGTGCAGGAGGCA	

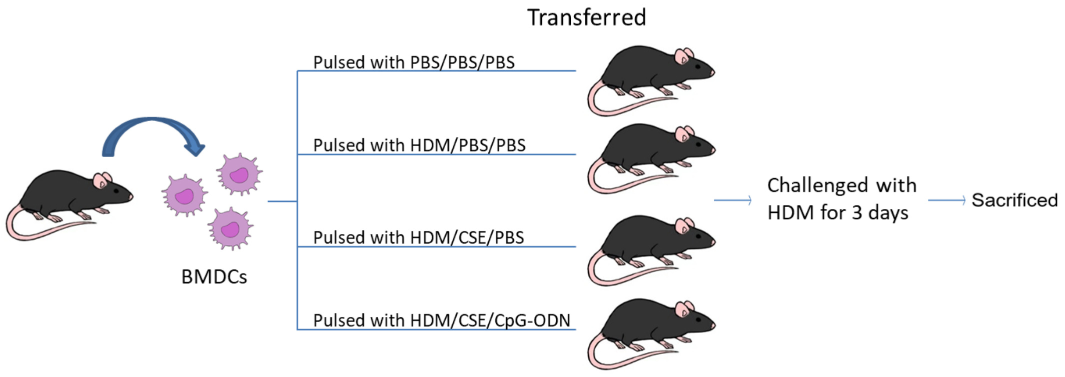

Figure S1. Experimental protocol for the study.

BMDCs were generated from the bone marrow of C57BL/6 mice. At day 8 of culture, BMDCs were pulsed overnight with PBS, HDM, CSE or CpG-ODN. At day 9, 1×10^6 cells were injected into the tracheas of anesthetized C57BL/6 recipients. On days 10–12 after adoptive transferred of BMDCs, the mice were challenged intranasally with HDM. On day 13, the animals were sacrificed for the acquisition of BALF, lung homogenate and lung sectioning.
